# Abnormal Glucose Tolerance Is Associated with a Reduced Myocardial Metabolic Flexibility in Patients with Dilated Cardiomyopathy

**DOI:** 10.1155/2016/3906425

**Published:** 2015-12-21

**Authors:** Domenico Tricò, Simona Baldi, Silvia Frascerra, Elena Venturi, Paolo Marraccini, Danilo Neglia, Andrea Natali

**Affiliations:** ^1^Dipartimento di Medicina Clinica e Sperimentale, Via Roma 67, 56126 Pisa, Italy; ^2^National Research Council, Institute of Clinical Physiology, Pisa, Italy

## Abstract

Dilated cardiomyopathy (DCM) is characterized by a metabolic shift from fat to carbohydrates and failure to increase myocardial glucose uptake in response to workload increments. We verified whether this pattern is influenced by an abnormal glucose tolerance (AGT). In 10 patients with DCM, 5 with normal glucose tolerance (DCM-NGT) and 5 with AGT (DCM-AGT), and 5 non-DCM subjects with AGT (N-AGT), we measured coronary blood flow and arteriovenous differences of oxygen and metabolites during *Rest*, *Pacing* (at 130 b/min), and *Recovery*. Myocardial lactate exchange and oleate oxidation were also measured. At *Rest*, DCM patients showed a reduced nonesterified fatty acids (NEFA) myocardial uptake, while glucose utilization increased only in DCM-AGT. In response to *Pacing*, glucose uptake promptly rose in N-AGT (from 72 ± 21 to 234 ± 73 nmol/min/g, *p* < 0.05), did not change in DCM-AGT, and slowly increased in DCM-NGT. DCM-AGT sustained the extra workload by increasing NEFA oxidation (from 1.3 ± 0.2 to 2.9 ± 0.1 *μ*mol/min/gO_2_ equivalents, *p* < 0.05), while DCM-NGT showed a delayed increase in glucose uptake. Substrate oxidation rates paralleled the metabolites data. The presence of AGT in patients with DCM exacerbates both the shift from fat to carbohydrates in resting myocardial metabolism and the reduced myocardial metabolic flexibility in response to an increased workload. This trial is registered with ClinicalTrial.gov NCT02440217.

## 1. Introduction 

The relationships between diabetes and impaired glucose tolerance (defined together as conditions of abnormal glucose tolerance, AGT) and dilated cardiomyopathy (DCM) are complex both at the clinical and the biochemical levels. Patients with AGT are more prone to develop both ischemic and nonischemic DCM [1–3] and derangements in glucose homeostasis are more prevalent among patients with DCM than in the general population [4]. The changes in whole-body metabolism, which are mainly secondary to insulin resistance and present in either AGT or DCM patients [5, 6], are expected to profoundly and similarly affect the heart, given the large reliance of myocardial metabolism on circulating substrates [7]. Despite this, the derangements in myocardial metabolism that have been described in the two conditions are opposite: while nonesterified fatty acids (NEFA) uptake and oxidation are reduced in primary DCM [8, 9], both are enhanced in diabetes [10], whereas glucose uptake and oxidation are depressed in diabetes and enhanced in DCM [9]. Therefore, particularly in DCM patients, the glucose tolerance status is expected to exert a relevant influence on myocardial metabolism and could justify the discrepancies of the data on myocardial metabolism in DCM [11]. Whether the above-mentioned changes contribute to the disease progression or are compensatory is unclear; while an excess uptake of NEFA would contribute to myocardial damage [12], a shift from lipids to carbohydrate would support myocardial energetics [13]. In addition to an abnormal metabolism in resting/fasting conditions, animal and human studies have demonstrated that both the failing myocardium and the diabetic myocardium display a reduced metabolic flexibility in response to substrate manipulations and to an increase in workload [12]. Therefore, chronic metabolic changes, which in certain conditions are adaptive, might render the myocardium unable to cope with stress. The changes in myocardial metabolism at baseline and in response to stress when impaired glucose metabolism and DCM are simultaneously present are unknown and might influence the process of (mal) adaptation. Interestingly, epidemiologic studies have suggested that diabetes does not worsen the prognosis of nonischemic DCM [14] and indeed in patients with DCM [15] higher fasting glucose levels are associated with a better prognosis; it is therefore possible that the opposing metabolic changes are somehow compensatory.

Our hypothesis is that the diabetic milieu attenuates the myocardial metabolic abnormalities of DCM, but it further reduces the already poor metabolic flexibility of the organ. To reduce the confounding effects of the systemic hormonal and substrate changes induced by either diseases and/or by their treatments, and also the variability induced by the different aetiology of DCM, we accurately selected only patients with NYHA class II/III idiopathic DCM and impaired glucose tolerance (IGT) or diet-controlled type 2 diabetes, with normal or impaired fasting glucose (<7.0 mmol/L).

## 2. Methods

The study was approved by the Ethics Committee of Institute of Clinical Physiology which is an autonomous institution of the National Research Council, and a written informed consent was obtained from each candidate on the day before cardiac catheterization after an exhaustive explanation of the protocol and its potential risks.

### 2.1. Study Population

We enrolled 5 patients with DCM and normal glucose tolerance (DCM-NGT) at the standard 75 g oral glucose tolerance test (OGTT), 5 patients with DCM and with either IGT or diabetes at the OGTT (DCM-AGT), and 5 patients with normal left ventricular function but with either IGT or diabetes at the OGTT (N-AGT). The subjects were preselected on the bases of clinical history, OGTT results, and left ventricular function, and they were admitted to the Cardiology Department of the NRC Institute of Clinical Physiology to undergo a diagnostic coronary angiography. If the subject had angiographically normal coronary arteries, then he/she was enrolled in the study. DCM was defined as left ventricular ejection fraction (LVEF) <40% and left ventricular end diastolic diameter (LVEDD) >56 mm. Exclusion criteria were NYHA class IV, atrial fibrillation, age ≥70 or ≤20 yrs, previous myocardial infarction, valvular heart disease, myocarditis or pericarditis, severe to moderate systemic arterial hypertension, fasting hyperglycaemia (>7.0 mmol/L) or treated diabetes, autoimmune diseases, neoplasia, and kidney, liver, or respiratory failure. N-AGT patients underwent coronary angiography because of either history of angina-like chest pain and/or previous stress tests suspicious for myocardial ischemia. Normal left ventricular function was defined as LVEF >50% and LVEDD <56 mm. All coronary-active drugs, including nitrates, Ca^++^-antagonists, *β*-blockers, and ACE-inhibitors, were suspended at least 24 hours before catheterization.

### 2.2. Cardiac Catheterization

A 6F guide catheter was placed in the left main coronary ostium through a 7F femoral artery introducer. An intracoronary heparin bolus (100 U/Kg) was given and a 0.014 Doppler flow wire (FloWire, Volcano Corp.) was advanced into the LAD. A 2.9 F, 10 MHz intravascular ultrasound (IVUS) catheter (Eagle Eye Gold, Volcano Corp.) was passed over the flow wire and positioned in the LAD immediately distal to the first septal perforating branch. The position of the IVUS catheter and flow wire was documented by angiography and was verified throughout the study by fluoroscopic control. After calibration, phasic and mean coronary perfusion pressure (from the guiding catheter) and coronary flow velocity signals were continuously recorded together with ECG (leads DI-DII-DIII). IVUS images were obtained for ≥30 seconds at each step of the protocol, with temporal synchronization with flow velocity signal, and recorded for offline analysis. After LAD instrumentation, a 5F catheter was advanced into the coronary sinus, up to the great cardiac vein, to withdraw venous blood from the LAD territory. Then, a unipolar pacing catheter was positioned into the right atrium. Arterial sampling was done from the 7F femoral artery introducer catheter.

### 2.3. Study Protocol

The study protocol consisted of three steps:* Rest*,* Pacing*, and* Recovery*. After the instrumentation was completed (about 30 min),* Rest* arteriovenous sampling was performed at times −15 min and 0 min. After 0 min, heart rate was increased by atrial pacing to 110 bpm for 3 minutes and to 130 bpm for 3 additional minutes and arteriovenous sampling was repeated at the end of each step (at times 3 and 6 min). Pacing was then stopped and at times 1, 5, 15, and 30 min into the* Recovery* period other pairs of arterial and venous samples were withdrawn. The first 5 mL of blood from each line was discarded to avoid contamination and then blood samples were transferred in* ad hoc* prepared tubes and kept in ice. In 4 N-AGT and in 5 DCM-AGT subjects, paired A-V blood samples were also collected in heparinised syringes and gas analysis immediately executed (Instrumentation Laboratory, Blood Gas Analyser IL 1620, Bedford, MA). In 5 N-AGT, in 4 DCM-AGT, and in 4 DCM-NGT, after the completion of diagnostic angiography, a bolus of 110 mg of [3-^13^C]-L-lactate and 50 *μ*Ci of [9,10^3^H]-oleate was followed by* i.v.* continuous infusion of [9,10^3^H]-oleate (84 *μ*Ci/h) and [3-^13^C]-L-lactate (130 mg/h) allowing a 30-minute equilibration period. The lactate stable isotope was used to measure net lactate uptake and net lactate release from the myocardium by the difference with the unlabelled lactate exchange. Labelled oleate was used to measure fractional oleate extraction and the fraction of oleate undergoing oxidation from the measurement of the myocardial ^3^H_2_O production [16].

### 2.4. Assays and Calculations

#### 2.4.1. Hemodynamics

Mean flow velocity was derived from the Doppler wire signal as the average time of spectral peak velocity. LAD lumen area (IVUS) was computed in systole and diastole for different cardiac cycles and then averaged. The absolute coronary blood flow (CBF) in the LAD was obtained by the formula

(1) CBFmL/min=mean  flow  velocitycm/sec∗LAD  lumen  areacm2∗60.

Since DCM and N-AGT patients had very different LV masses, CBF values were corrected for the estimated LAD-dependent myocardial mass using the following formula:

(2) $$\begin{array}{c} \text{CBF}\left(\;m/min/g\;\right)=\frac{\text{CBF}\left(mL/min\right)}{\left(0.54*\text{LVmass}\left(g\right)\right)}. \end{array}$$

The fraction of LV mass putatively supplied by the LAD (0.54) was chosen based on the following considerations. First, in noninvasive wall motion or perfusion studies, 8–12 out of 17 LV segments (43–65%, average 54%) are attributed to LAD [17, 18]. Second, in 6 DCM patients of our population, absolute blood flow in the LAD territory (mL/min/g) was independently measured by PET and ^13^N-ammonia, and the agreement between the directly measured and the calculated values was good (0.61 ± 0.18 versus 0.56 ± 0.13 mL/min/g, *p* = ns).

#### 2.4.2. Metabolites

Whole blood glucose, lactate and *β*-OH-butyrate, pyruvate, glycerol and alanine, and serum triglycerides were determined spectrophotometrically on a Beckman Synchron CX7 analyser (Global Medical Instrumentation, Ramsey, MN, USA). Whole blood samples were collected into iced tubes containing 1 M perchloric acid and the supernatant was stored at –20°C and assayed within 30 days. NEFA concentration was determined in plasma after centrifugation of EDTA-treated blood samples using a colorimetric assay (Wako Chemicals Gmbh, Neuss, Germany).

#### 2.4.3. Isotopes

The isotopic enrichment of lactate with [^13^C]-L-lactate was measured by gas chromatography-mass spectrometry in plasma samples deproteinized with perchloric acid and derivatized using* N*-methyl-*N*-(*t*-butyldimethylsilyl)-trifluoroacetamide (Sigma, St. Louis, MO, USA). Plasma [9,10^3^H]-oleate concentration was measured by extracting fatty acids from 0.5 mL of plasma in 3 mL of HCl-heptane-isopropanol (1 : 10 : 40) and counting the radioactivity of the organic phase with a liquid scintillation counter (model LS 6500; Beckman) while ^3^H_2_O concentration, derived from [9,10-^3^H] oleate oxidation, was measured by counting the water phase. Myocardial substrate uptake, release, and oxidation were calculated as the arteriovenous concentration difference times CBF as previously described [19].

#### 2.4.4. Gas Analysis and Oxygen Equivalent Calculations

Total O_2_ and CO_2_ blood content were calculated as previously described [20]. Carbohydrate and lipid oxidation rates and energy expenditure were calculated using standard equation for indirect calorimetry as explained in detail in [21]. Substrates oxygen equivalents were calculated on the bases of the predicted ATP yields generated by the complete oxidation of each glucose, lactate, *β*-OH-butyrate, and oleate molecule taken up by the myocardial tissue (36, 12, 24, and 131 per mole, resp.) that was converted in oxygen according to the substrate specific ATP/O_2_ ratio (oleate: 5.6 mol/mol; glucose, lactate, and *β*-OH-butyrate: 6 mol/mol) [21]. Values were averaged over the 3 study periods using the following samples:* Rest* (−10 and 0 min),* Pacing* (3 and 6 min), and* Recovery* (11, 16, and 31 min).

#### 2.4.5. Statistics

Data are presented as mean ± SEM. Differences in mean and prevalence were tested with the Wilcoxon/Kruskal-Wallis and *Chi* ^2^ tests, respectively. Differences in coronary hemodynamic and metabolic data were compared between study conditions (*Rest, Pacing, and Recovery*) by one-way ANOVA for repeated measure and within each study condition between groups by two-way ANOVA for single repeated measure over time and the least square mean estimates ± SEM are given. *p* value less than 0.05 was considered significant.

## 3. Results

### 3.1. Clinical Characteristics of the Study Populations

The clinical characteristics of the 3 study groups are shown in Table 1. The groups did not significantly differ in terms of age, gender distribution, BMI, fasting glucose, and fasting insulin. History of angina-like chest pain was more frequent in N-AGT groups. DCM patients showed symptoms and echocardiographic and serum indices (NT-proBNP and BNP) and drug treatments, suggesting a non-severely compromised LV systolic function which was of similar degree in AGT and NGT. The 2 groups of AGT subjects, N and DCM, had similar fasting and 2 h OGTT glucose values, while DCM subjects showed higher fasting serum triglycerides.

### 3.2. Coronary Haemodynamic

**Figure 1:**
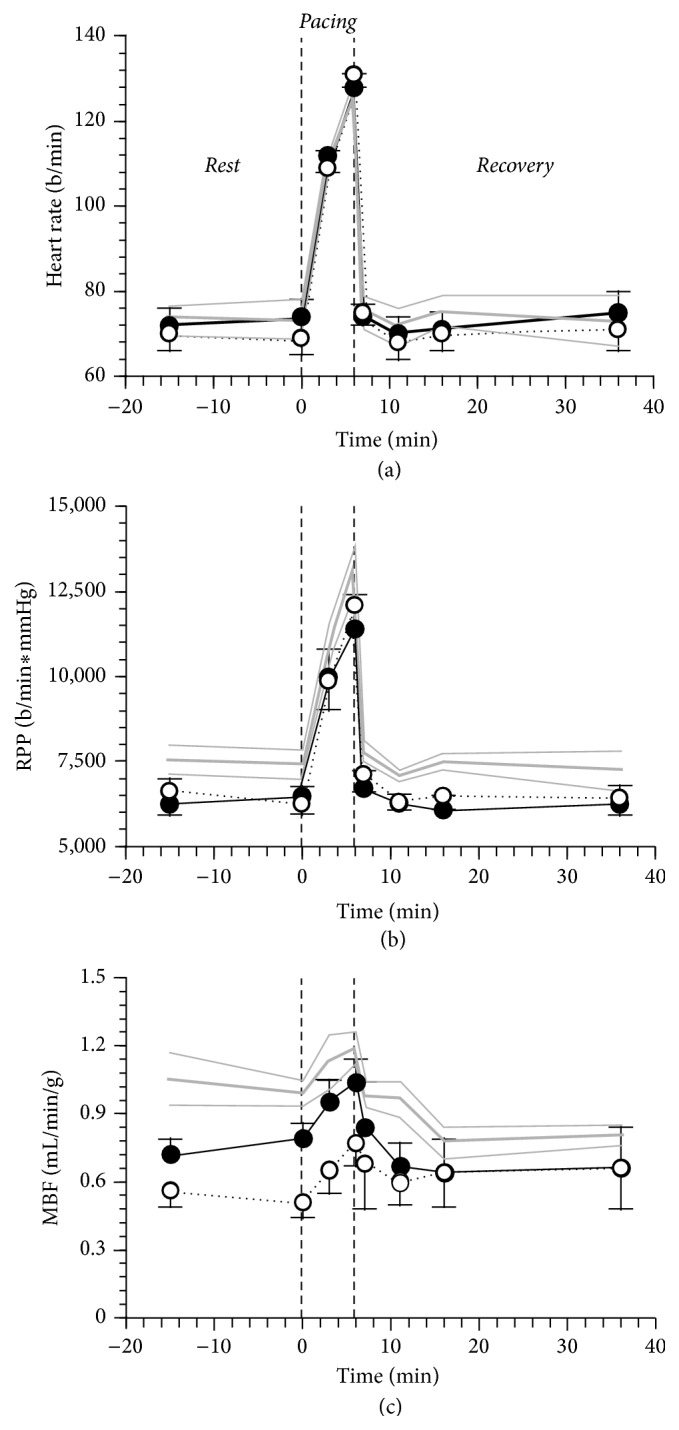
Heart rate, rate pressure product (RPP), and myocardial blood flow (MBF) in 5 N-AGT (gray line), 5 DCM-NGT (dotted black line), and 5 DCM-AGT (continuous black line) in the 3 phases of the study protocol:* Rest*,* Pacing*, and* Recovery*. The plotted values are mean ± SEM. The thin gray lines represent the SEM of the N-AGT group.

Data on coronary hemodynamic during and after pacing stress are summarized in Figure 1. While heart rates were superimposable in the 3 study groups, rate pressure product tended to be higher in N-AGT (*p* = 0.11), with mean blood pressure being significantly higher in this study group, with respect to DCM-AGT and NGT patients (107 ± 4* versus *87 ± 5 and 90 ± 4 mmHg, *p* < 0.01), throughout the study. Coronary specific blood flow in the LAD territory, during both* Rest* and* Pacing*, was lower in DCM-NGT than in N-AGT (*p* < 0.05) with the values in DCM-AGT being intermediate, though not statistically different from the other groups. In the* Recovery*, coronary blood flow was similar in the 3 study groups; however, with respect to* Rest*, N-AGT reached blood flow values that were significantly lower (*p* < 0.05), while the other 2 study groups returned to baseline.

### 3.3. Myocardial Metabolism and Energetics

Arterial whole blood concentrations of lactate (0.565 ± 0.074* versus *0.515 ± 0.073* versus *0.697 ± 0.070 mmol/L) and glucose (4.6 ± 0.2* versus *4.9 ± 0.2* versus *4.9 ± 0.2 mmol/L) were similar in N-AGT* versus* DCM-NGT* versus* DCM-AGT, whereas whole blood *β*-OH-butyrate was higher in DCM-NGT (0.272 ± 0.078* versus *0.704 ± 0.202* versus *0.139 ± 0.043 mmol/L) and plasma NEFA was higher in N-AGT (1.758 ± 0.105* versus *1.178 ± 0.158* versus *1.412 ± 0.149 mmol/L) (*p* < 0.02 for both). None of these arterial concentrations changed during the study.

**Figure 2:**
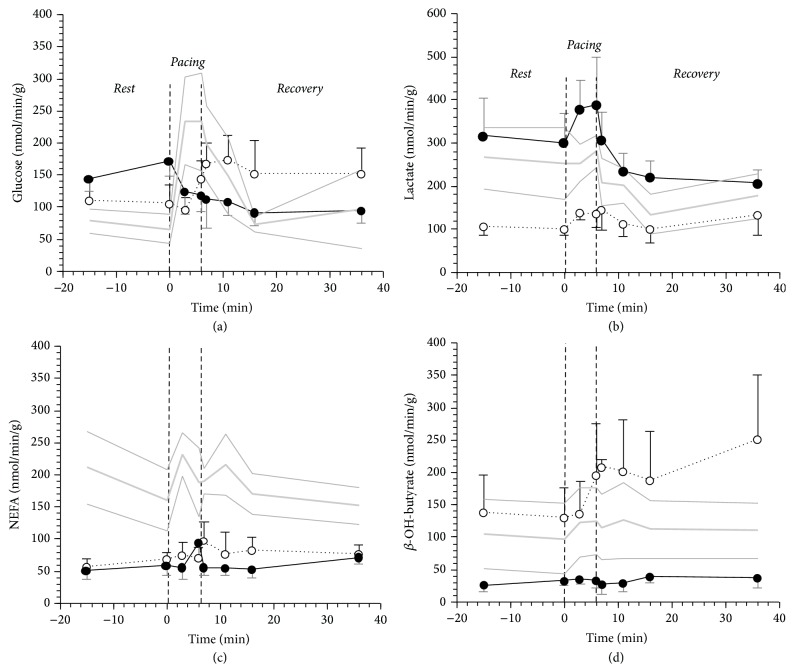
Cardiac glucose (a), lactate (b), NEFA (c), and *β*-OH-butyrate (d) uptake in 5 N-AGT (gray line), 5 DCM-NGT (dotted black line), and 5 DCM-AGT (continuous black line) in the 3 phases of the study protocol:* Rest*,* Pacing*, and* Recovery.* The plotted values are mean ± SEM. The thin gray lines represent the SEM of the N-AGT group.

With respect to N-AGT, the patients with DCM (NGT and AGT altogether) in* Rest* showed a 60% reduction in myocardial NEFA uptake 60% (59 ± 10* versus *186 ± 18 nmol/min/g, *p* = 0.001) and a doubling of myocardial glucose uptake (135 ± 15* versus *73 ± 8 nmol/min/g, *p* = 0.013) (Figure 2). Although glucose uptake was not statistically different between DCM-NGT and DCM-AGT, the increase with respect to N-AGT was more pronounced (+120%) in the latter than in the former (+48%). The reduced NEFA uptake was not determined by a lower extraction rate (14 ± 2* versus *16 ± 2%, *p* = ns) but by the combination of lower arterial NEFA (1.944 ± 0.155* versus *1.251 ± 0.129 mmol/L, *p* < 0.0001) and lower myocardial blood flow (0.89 ± 0.08* versus *0.71 ± 0.07 mL/min/g, *p* = 0.03). Similar NEFA extraction was confirmed by labelled oleate data (31 ± 3* versus *35 ± 3%) that, in absolute values, were also higher than the estimates obtained with natural substrates as previously reported [22]. Oleate and ^3^H_2_O data also indicated a similar fractional oxidation rate in normal and DCM patients (66 ± 7* versus *64 ± 6%).

In* Rest*, lactate uptake was particularly low in DCM-NGT and *β*-OH-butyrate uptake was particularly low in DCM-AGT with respect to the other study groups (Figure 2). Neither glycerol nor alanine nor triglycerides net balances were significantly different from zero (Table 2). Pyruvate uptake, although still quantitatively minute (10% of lactate uptake), was not different from zero in DCM-AGT, while it was null in DCM-NGT.

**Figure 3:**
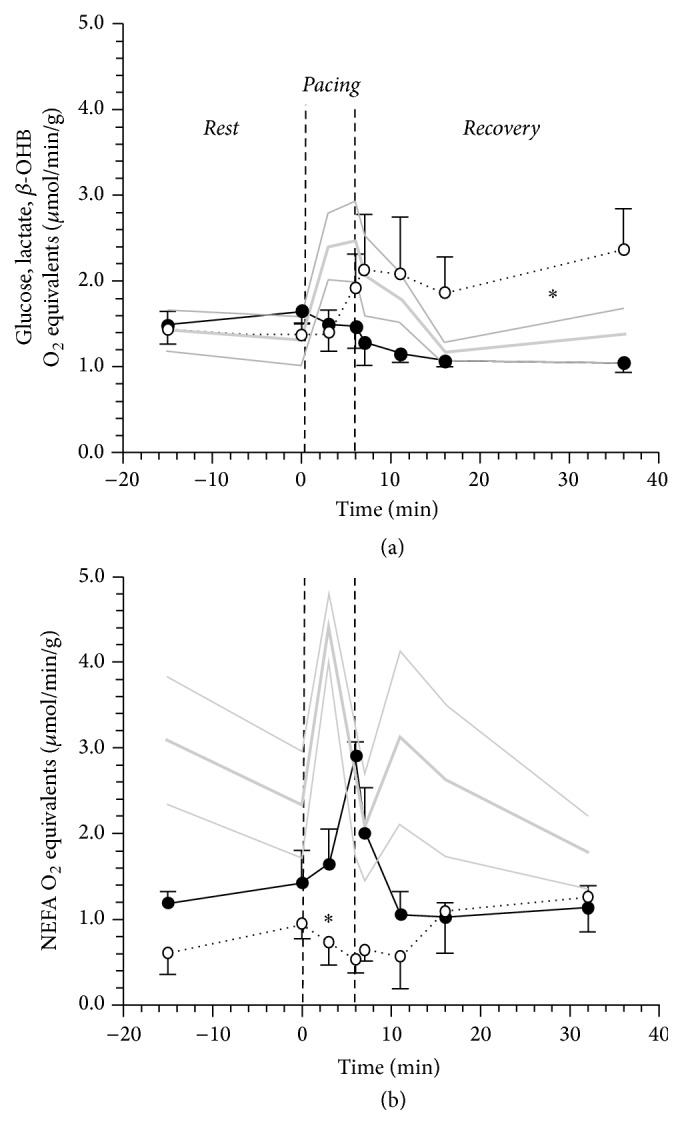
Cardiac energy generation expressed on oxygen equivalents from (a) small molecules, blood born, readily oxidizable substrates (calculated summing up glucose, lactate, and *β*-OH-butyrate net balances and assuming their full oxidation) as compared to energy generated by NEFA oxidation as measured from [9,10^3^H]-oleate conversion to ^3^H_2_O in 5 N-AGT (gray line), 4 DCM-NGT (dotted black line), and 4 DCM-AGT (continuous black line) in the 3 phases of the study protocol:* Rest*,* Pacing*, and* Recovery*. The plotted values are mean ± SEM. The thin gray lines represent the SEM of the N-AGT group. *∗* indicates a *p* < 0.05 for the comparison between DCM-AGT and DCM-NGT over the study phase by 2-way ANOVA for repeated measures.

In response to pacing, DCM-NGT showed a delayed and persistent increase in glucose, NEFA, and *β*-OH-butyrate uptake, while DCM-AGT showed a short lasting increment only in NEFA and lactate coupled with a reduction in glucose and no change in *β*-OH-butyrate uptake (Figure 2). Once we compared the contribution of the readily oxidised, small size, high respiratory quotient substrates (glucose, lactate, and *β*-OH-butyrate) with the NEFA oxidation rates measured by tracer, we observed that DCM-NGT paid the extra work induced by pacing increasing the utilization of small molecules, mostly in the* Recovery* period, while DCM-AGT sustained the myocardial work by a short lasting rise in NEFA oxidation being unable to modify the utilization of the other substrates (Figure 3). The N-AGT subjects fuelled the pacing with a prompt increase in glucose and small molecules uptake that persisted 15 min into the* Recovery* period. Interestingly, the rise in NEFA oxidation in DCM-AGT patients was determined by a rise in both NEFA net balance (from 54 ± 32 to 75 ± 29 nmol/min/g, *p* < 0.05) and fractional oxidation (from 72 ± 8 to 93 ± 9, *p* < 0.05) as estimated from oleate conversion into ^3^H_2_O. Neither glycerol nor alanine nor pyruvate nor triglycerides net balances were significantly different from* Rest* during* Pacing* or* Recovery *(Table 2).

The metabolic inflexibility of DCM-AGT patients is confirmed also by myocardial gas exchange data that shows that the extra cardiac work is almost entirely supported by lipid, whilst in N-AGT it is supported by carbohydrates oxidation (Table 3).

**Figure 4:**
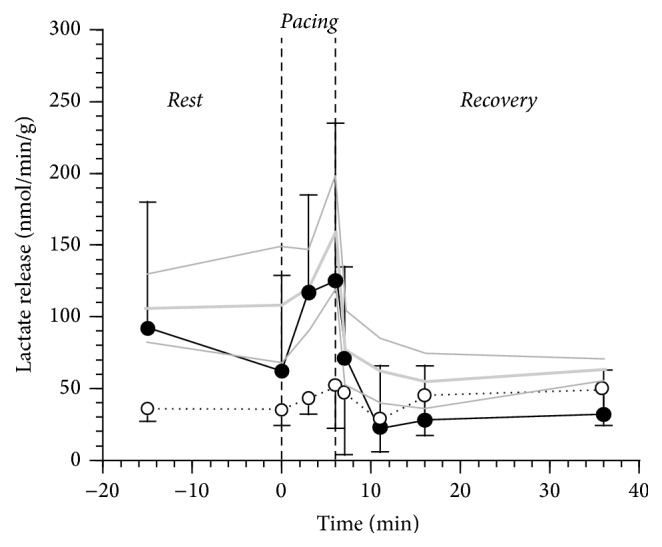
Cardiac lactate release as estimated subtracting [3-^13^C]-L-lactate cardiac uptake to the natural substrate net balance in 5 N-AGT (gray line), 4 DCM-NGT (dotted black line), and 4 DCM-AGT (continuous black line) in the 3 phases of the study protocol:* Rest*,* Pacing*, and* Recovery*. The plotted values are mean ± SEM. The thin gray lines represent the SEM of the N-AGT group.

The myocardial release of lactate estimated by tracer was only minimally stimulated by pacing but promptly returned to baseline values, suggesting no evidence of metabolic ischemia in either study group (Figure 4).

## 4. Discussion

We confirm that patients with DCM have a reduced myocardial NEFA uptake and oxidation, a tendency to an enhanced myocardial glucose utilization, and a reduced metabolic flexibility to face an extra energy demand [8]. The novel aspect is that abnormal glucose tolerance does not modify resting heart metabolism but is associated with a more severe metabolic inflexibility that involves other metabolites: while in NGT we observed a delay in the shift to glucose and other small molecules (lactate and *β*-OH-butyrate) utilization, in AGT patients the use of small molecules resulted to be fixed and the extra work was done entirely relying upon NEFA.

We also found that the reduction in NEFA uptake is essentially caused by a reduced substrate supply (i.e., blood flow and arterial concentration) and not by an impaired substrate extraction. This finding was confirmed by the tracer exchange data that revealed similar extraction rates in N and DCM despite the different net balances. The discrepancy between the tracer and the natural substrate estimated tissue extraction, which has already been observed and extensively discussed [22], was similar in N-AGT, DCM-NGT, and DCM-AGT as can be appreciated comparing the slope values yielded by regression analysis (forced to a 0 intercept) on the two independent estimates within each study group (1.55 ± 0.12, 1.46 ± 0.17, and 1.46 ± 0.16, resp.). Similar NEFA and [^2^H_2_]-palmitate extraction rates have also recently been reported in DCM patients and type 2 diabetic patients with normal left ventricular function [23]; however, having not measured blood flow but only oxygen uptake, the authors could not detect the presence of absolute reduction in metabolic fluxes in DCM patients. The overall similarity between DCM-NGT and DCM-AGT in resting conditions is against our hypothesis that abnormal glucose metabolism would oppose the chronic metabolic shift of DCM patients. The presence of DCM imposes a change to myocardial metabolism that prevails on the consequences of a systemic derangement in glucose metabolism. The less severe alteration in glucose and lactate uptake in DCM-NGT suggest that the enhanced carbohydrate utilization is peculiar of patients with DCM and AGT and this might explain why these findings are not consistent in the literature. The resting lower rates of *β*-OH-uptake in DCM-AGT are probably only the consequence of the lower arterial levels of the substrate.

Myocardial metabolic response to stress in DCM patients with and without AGT had not previously been investigated. In normal hearts, high rate pacing induces a rapid increase in glucose and lactate uptake coupled with a relative decline in NEFA uptake [24, 25]. Clinical and experimental studies suggest that this response is advantageous and increases myocardial mechanical efficiency as carbohydrates are more efficient substrates than lipids since they provide more energy for any given amount of oxygen consumed [26–28]. The metabolic response to pacing in DCM patients was different according to glucose tolerance status: DCM-AGT showed a complete inability to shift to glucose and/or small molecules utilization, and in DCM-NGT the shift was delayed. The inflexibility of DCM-AGT was also confirmed by gas analysis data that yielded estimates that were very close to natural substrate and tracer-derived data. Interestingly, these data also indicate that both* Rest* and* Pacing* oxygen uptake (and energy expenditure) per g of tissue are approximately 40–50% lower in DCM patients suggesting a reduced myocardial energetics density (per g of tissue); however, the small sample size and the lack of accurate measurements of cardiac work prevent from quantitatively comparing the energetic efficiency in the two groups. The normal aerobic metabolism (i.e., lack of the metabolic fingerprints of ischemia) is also independently and directly confirmed by lactate tracer-derived release data during either* Pacing* or* Recovery* in each and all study groups (Figure 4). The presence of AGT therefore worsens the already impaired metabolic flexibility observed in DCM patients. The inability to use high respiratory quotient, readily oxidizable, blood born small molecules to sustain a rapid increase in energy demand is rather surprising particularly in a tissue where the oxidation of the major alternative substrate (NEFA) is depressed and is also more demanding in terms of oxygen. One possible explanation is that in DCM-AGT NEFA oxidation can increase because there is an increase in myocardial blood flow that is less evident in DCM-NGT patients who, therefore, must rely upon alternative blood born substrates. Clearly, there must be other explanations since we also observed a more efficient oleate fractional oxidation in DCM-AGT during pacing. One of the possible mechanisms explaining the metabolic inflexibility of the failing heart is the paradoxical downregulation of key enzymes of the carbohydrate oxidative pathway, in spite of a higher glucose oxidation rate, as it has previously been described in dogs [29].

Given the pathophysiologic nature of the question addressed and of the methods employed, the clinical consequences of our findings remain entirely speculative. The lack of flexibility, if confirmed, would expose the myocardium to a lesser energetic efficiency (higher oxygen cost for NEFA oxidation rather than small molecules) and potentially to a dangerous reliance on plasma NEFA availability, whose plasma concentration, for example, is dramatically reduced in the postabsorptive state. In the long term, it would be important to understand whether the different responses to pacing observed in AGT and NGT are adaptative or maladaptative and/or whether they modify the natural course of the myocardial disease. This might influence the clinical response to drugs that directly shift myocardial substrate utilization from NEFA to glucose (e.g., trimetazidine and ranolazine) and also to drugs like beta blockers that are able to interfere with the changes in systemic and myocardial metabolism induced by catecholamines and eventually by the presence or absence of ischemia [30].

The small number of subjects per group, due to the complexity of the experimental procedure, is a potential limitation of this study. However, our main hypothesis that an impaired glucose metabolism might prevent the metabolic changes described in the myocardium of patients with DCM can be rejected with a high level of confidence having observed in AGT patients, if any, a more severe metabolic derangement. The use of pacing as a tool to stimulate myocardial metabolism is a limitation since in physiology the extra cardiac work is normally associated with hormonal (essentially catecholamines) and substrate changes (NEFA mobilization); however, our choice was dictated by the need of constant plasma substrates concentration (required for the use of metabolic tracers) and by the constraints of the Catheterization Laboratory setting. The lack of a control group with normal glucose metabolism and normal heart function is also a limitation of our study. However, this choice was related to the fact that these subjects are very rare among those who undergo a coronary angiography and, most importantly, our major concern was to avoid attributing the differences observed in myocardial metabolism between AGT and NGT patients with DCM to the presence of AGT, whose impact on myocardial metabolism has not been carefully characterised.

## 5. Conclusions

The presence of mild derangements in glucose metabolism (IGT and early diabetes) exacerbates the metabolic shift from fat to carbohydrates that occurs at rest in the myocardium of patients with dilated cardiomyopathy and also the already poor metabolic flexibility of the tissue in facing an acute myocardial workload increase. This might render the myocardium less efficient and/or more vulnerable and might modify its response to therapies that interfere with glucose metabolism.

## Figures and Tables

**Table 1:** Clinical characteristics of the study populations.

	N-AGT	DCM-NGT	DCM-AGT
*N*	5	5	5
Age (year)	62 ± 6	55 ± 3	60 ± 6
Males/females (*n*)	3/2	4/1	3/2
Angina (*n*)	5	2	1
NYHA class II/III (*n*)	1/0	3/2^*∗*^	3/2^*∗*^
LV mass (g)	175 ± 16	288 ± 27^*∗*^	292 ± 43^*∗*^
LVEF (%)	56 ± 2	34 ± 2^*∗*^	31 ± 1^*∗*^
LVEDD (mm)	51 ± 2	64 ± 2^*∗*^	66 ± 2^*∗*^
BNP (pg/mL)	20 ± 7	98 ± 22^*∗*^	104 ± 30^*∗*^
NT-proBNP (pg/mL)	128 ± 37	598 ± 130^*∗*^	604 ± 127^*∗*^
Body mass index (kg/m^2^)	28 ± 2	29 ± 2	30 ± 2
Fasting insulin (pmol/L)	77 ± 8	69 ± 9	84 ± 10
Fasting glucose (mmol/L)	5.6 ± 0.2	5.4 ± 0.2	5.8 ± 0.2
OGTT 2 h glucose (mmol/L)	10.8 ± 1.1	6.0 ± 0.6^*∗*^	10.9 ± 0.7^§^
Triglycerides (mmol/L)	0.66 ± 0.10	0.65 ± 0.19	1.14 ± 0.20^§^
*β*-blockers (*n*)	3	5	5
ACE-inhibitors/AT-1 antagonists (*n*)	4	5	5
Antialdosteronic agents (*n*)	0	3^*∗*^	4^*∗*^
Furosemide ≥25 mg/die (*n*)	1	5^*∗*^	5^*∗*^

LV mass: left ventricular mass; LVEF: left ventricular ejection fraction; LVEDD: left ventricular end diastolic diameter.

^*∗*^ *p* < 0.05 for the comparison with N-AGT.

^§^ *p* < 0.05 for the comparison between DCM-NGT and AGT.

**Table 2:** Cardiac net balances of other metabolites.

		N-AGT	DCM-NGT	DCM-AGT	*p*
Alanine (nmol/min/g)	*Rest*	−17.4 ± 6.3	−7.9 ± 3.4	−7.5 ± 7.6	ns
	*Pacing*	−16.8 ± 6.8	−14.1 ± 4.2	−8.8 ± 5.2	
	*Recovery*	−18.2 ± 8.1	−5.1 ± 6.7	−8.0 ± 3.1	

Pyruvate (nmol/min/g)	*Rest*	12.9 ± 5.8	3.9 ± 2.4	24.8 ± 6.1	<0.05
	*Pacing*	14.1 ± 5.0	3.8 ± 1.9	32.4 ± 8.1	
	*Recovery*	14.6 ± 4.7	−1.9 ± 2.8	22.6 ± 3.8	

Glycerol (nmol/min/g)	*Rest*	−0.3 ± 3.4	2.8 ± 2.5	1.6 ± 2.9	ns
	*Pacing*	−0.1 ± 3.9	−4.6 ± 5.1	−5.5 ± 8.9	
	*Recovery*	−2.1 ± 2.6	−1.6 ± 3.9	−2.0 ± 1.9	

Triglycerides (nmol/min/g)	*Rest*	1.7 ± 5.4	6.0 ± 2.8	3.7 ± 7.1	ns
	*Pacing*	7.6 ± 4.1	3.5 ± 5.6	−4.6 ± 5.1	
	*Recovery*	7.1 ± 7.4	7.0 ± 4.3	4.2 ± 3.2	

Metabolite concentrations were available for all patients: 5 N-AGT, 5 DCM-NGT, and 5 DCM-AGT.

*p* values refer to the comparison between N-AGT and DCM-AGT as estimated by 2-way ANOVA for one repeated measure over time.

**Table 3:** Gas analysis data.

	Phase	N-AGT	DCM-AGT	*p*
MVO_2_ (*μ*mol/min/g)	*Rest*	5.7 ± 0.6	4.1 ± 0.6	ns
	*Pacing*	6.5 ± 0.6^*∗*^	5.1 ± 0.4^*∗*^	
	*Recovery*	5.1 ± 0.4	3.4 ± 0.2	

RQ	*Rest*	0.80 ± 0.05	0.80 ± 0.10	<0.05
	*Pacing*	0.82 ± 0.03	0.77 ± 0.05	
	*Recovery*	0.85 ± 0.04	0.88 ± 0.10	

Carbohydrates oxidation (nmol/min/g)	*Rest*	402 ± 83	230 ± 145	<0.02
	*Pacing*	767 ± 58^*∗*^	208 ± 67	
	*Recovery*	450 ± 102	362 ± 103	

Lipids oxidation (nmol/min/g)	*Rest*	160 ± 73	83 ± 32	<0.02
	*Pacing*	179 ± 44	159 ± 35^*∗*^	
	*Recovery*	101 ± 20	62 ± 33	

EE from carbohydrates (cal/min/g)	*Rest*	0.26 ± 0.13	0.15 ± 0.08	<0.02
	*Pacing*	0.51 ± 0.04^*∗*^	0.14 ± 0.06	
	*Recovery*	0.30 ± 0.09	0.24 ± 0.09^*∗*^	

EE from lipids (cal/min/g)	*Rest*	0.38 ± 0.09	0.20 ± 0.10	<0.02
	*Pacing*	0.43 ± 0.11	0.38 ± 0.08^*∗*^	
	*Recovery*	0.33 ± 0.09	0.21 ± 0.08	

Gas analysis data were available for a subset of patients: 4 DCM-NGT and 4 DCM-AGT.

*p* values refer to the comparison between N-AGT and DCM-AGT as estimated by 2-way ANOVA for one repeated measure over time.

^*∗*^ *p* < 0.05 for the comparison with the preceding study protocol step (*Pacing* versus *Rest* and *Recovery* versus *Pacing*).

MVO_2_: myocardial oxygen uptake; RQ: respiratory quotient; EE: energy expenditure.

## Abbreviations

AGT: Abnormal glucose tolerance
CBF: Coronary blood flow
DCM: Dilated cardiomyopathy
EE: Energy expenditure
IGT: Impaired glucose tolerance
IVUS: Intravascular ultrasound
LAD: Left anterior descending artery
MBF: Myocardial blood flow
MVO_2_: Myocardial oxygen uptake
NEFA: Nonesterified fatty acids
NGT: Normal glucose tolerance
OGTT: Oral glucose tolerance test
RPP: Rate pressure product
RQ: Respiratory quotient.

## References

[1] Kannel W. B., Hjortland M., Castelli W. P. (1974). Role of diabetes in congestive heart failure: the Framingham study. *The American Journal of Cardiology*.

[2] Bertoni A. G., Hundley W. G., Massing M. W., Bonds D. E., Burke G. L., Goff D. C. (2004). Heart failure prevalence, incidence, and mortality in the elderly with diabetes. *Diabetes Care*.

[3] Nichols G. A., Gullion C. M., Koro C. E., Ephross S. A., Brown J. B. (2004). The incidence of congestive heart failure in type 2 diabetes: an update. *Diabetes Care*.

[4] Bertoni A. G., Tsai A., Kasper E. K., Brancati F. L. (2003). Diabetes and idiopatihic cardiomyopathy: a nationwide case-control study. *Diabetes Care*.

[5] AlZadjali M. A., Godfrey V., Khan F. (2009). Insulin resistance is highly prevalent and is associated with reduced exercise tolerance in nondiabetic patients with heart failure. *Journal of the American College of Cardiology*.

[6] Ashrafian H., Frenneaux M. P., Opie L. H. (2007). Metabolic mechanisms in heart failure. *Circulation*.

[7] Stanley W. C., Recchia F. A., Lopaschuk G. D. (2005). Myocardial substrate metabolism in the normal and failing heart. *Physiological Reviews*.

[8] Neglia D., de Caterina A., Marraccini P. (2007). Impaired myocardial metabolic reserve and substrate selection flexibility during stress in patients with idiopathic dilated cardiomyopathy. *The American Journal of Physiology—Heart and Circulatory Physiology*.

[9] Dávila-Román V. G., Vedala G., Herrero P. (2002). Altered myocardial fatty acid and glucose metabolism in idiopathic dilated cardiomyopathy. *Journal of the American College of Cardiology*.

[10] Peterson L. R., Herrero P., Schechtman K. B. (2004). Effect of obesity and insulin resistance on myocardial substrate metabolism and efficiency in young women. *Circulation*.

[11] Lopaschuk G. D., Ussher J. R., Folmes C. D. L., Jaswal J. S., Stanley W. C. (2010). Myocardial fatty acid metabolism in health and disease. *Physiological Reviews*.

[12] Larsen T. S., Aasum E. (2008). Metabolic (in)flexibility of the diabetic heart. *Cardiovascular Drugs and Therapy*.

[13] Turer A. T., Malloy C. R., Newgard C. B., Podgoreanu M. V. (2010). Energetics and metabolism in the failing heart: important but poorly understood. *Current Opinion in Clinical Nutrition and Metabolic Care*.

[14] Domanski M., Krause-Steinrauf H., Deedwania P. (2003). The effect of diabetes on outcomes of patients with advanced heart failure in the BEST trial. *Journal of the American College of Cardiology*.

[15] Issa V. S., Amaral A. F., Cruz F. D. (2010). Glycemia and prognosis of patients with chronic heart failure—subanalysis of the long-term prospective randomized controlled study using repetitive education at six-month intervals and monitoring for adherence in heart failure outpatients (remadhe) trial. *American Heart Journal*.

[16] Osorio J. C., Stanley W. C., Linke A. (2002). Impaired myocardial fatty acid oxidation and reduced protein expression of retinoid x receptor-*α* in pacing-induced heart failure. *Circulation*.

[17] Cerqueira M. D., Weissman N. J., Dilsizian V. (2002). Standardized myocardial segmentation and nomenclature for tomographic imaging of the heart: A Statement for Healthcare Professionals from the Cardiac Imaging Committee of the Council on Clinical Cardiology of the American Heart Association. *Circulation*.

[18] Pereztol-Valdés O., Candell-Riera J., Santana-Boado C. (2005). Correspondence between left ventricular 17 myocardial segments and coronary arteries. *European Heart Journal*.

[19] Recchia F. A., Osorio J. C., Chandler M. P. (2002). Reduced synthesis of NO causes marked alterations in myocardial substrate metabolism in conscious dogs. *American Journal of Physiology—Endocrinology and Metabolism*.

[20] Ferrannini E., Santoro D., Bonadonna R., Natali A., Parodi O., Camici P. G. (1993). Metabolic and hemodynamic effects of insulin on human hearts. *The American Journal of Physiology—Endocrinology & Metabolism*.

[21] Ferrannini E. (1988). The theoretical bases of indirect calorimetry: a review. *Metabolism*.

[22] Wisneski J. A., Gertz E. W., Neese R. A., Mayr M. (1987). Myocardial metabolism of free fatty acids. Studies with 14C-labeled substrates in humans. *The Journal of Clinical Investigation*.

[23] Funada J., Betts T. R., Hodson L. (2009). Substrate utilization by the failing human heart by direct quantification using arterio-venous blood sampling. *PLoS ONE*.

[24] Camici P., Marraccini P., Marzilli M. (1989). Coronary hemodynamics and myocardial metabolism during and after pacing stress in normal humans. *The American Journal of Physiology: Endocrinology and Metabolism*.

[25] Bagger J. P., Thomassen A., Nielsen T. T. (2000). Cardiac energy metabolism in patients with chest pain and normal coronary angiograms. *The American Journal of Cardiology*.

[26] Burkhoff D., Weiss R. G., Schulman S. P., Kalil-Filho R., Wannenburg T., Gerstenblith G. (1991). Influence of metabolic substrate on rat heart function and metabolism at different coronary flows. *American Journal of Physiology—Heart and Circulatory Physiology*.

[27] Mazumder P. K., O'Neill B. T., Roberts M. W. (2004). Impaired cardiac efficiency and increased fatty acid oxidation in insulin-resistant ob/ob mouse hearts. *Diabetes*.

[28] Carley A. N., Severson D. L. (2005). Fatty acid metabolism is enhanced in type 2 diabetic hearts. *Biochimica et Biophysica Acta—Molecular and Cell Biology of Lipids*.

[29] Lei B., Lionetti V., Young M. E. (2004). Paradoxical downregulation of the glucose oxidation pathway despite enhanced flux in severe heart failure. *Journal of Molecular and Cellular Cardiology*.

[30] An D., Rodrigues B. (2006). Role of changes in cardiac metabolism in development of diabetic cardiomyopathy. *The American Journal of Physiology—Heart and Circulatory Physiology*.

